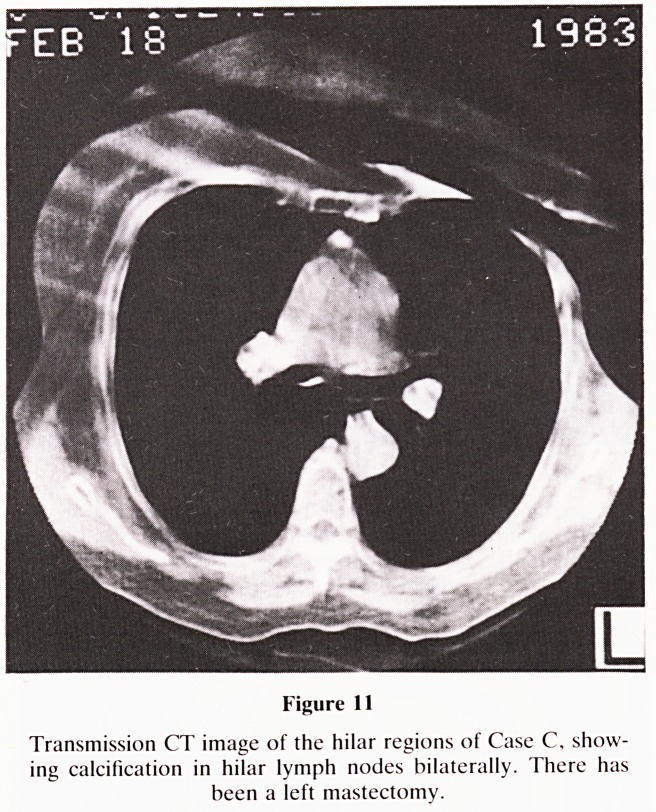# Pulmonary Uptake of MDP

**Published:** 1990-06

**Authors:** P. Goddard, J. Bell, P. Jackson, E. Pitcher, J. A. Bullimore, E. R. Davies

**Affiliations:** The Imaging Research Unit, Bristol Royal Infirmary; The Imaging Research Unit, Bristol Royal Infirmary; The Imaging Research Unit, Bristol Royal Infirmary; The Imaging Research Unit, Bristol Royal Infirmary; The Imaging Research Unit, Bristol Royal Infirmary; The Imaging Research Unit, Bristol Royal Infirmary


### Pulmonary uptake of MDP: Demonstration of site by correlation of emission and transmission computed tomography

**P. Goddard, J. Bell, P. Jackson, E. Pitcher, J. A. Bullimore, E. R. Davies**
The Imaging Research Unit, Bristol Royal Infirmary

### PULMONARY UPTAKE OF MDP

Radionuclide bone scanning is now a standard technique in the staging of many malignancies. Probably the most frequent application is in screening patients with known primary malignancy and potential bone metastases. In primary malignant bone tumours, bone scanning is also useful with images often showing the lesion to be more extensive than was suspected from radiographs (McKillop et al., 1974). Bone forming metastases in the lungs can sometimes be detected before they become visible on the chest radiograph (McKillop et al. 1974, Robinson 1979, Ghaed et al. 1981). Although radionuclide imaging of bone is very sensitive, it is of low specificity and a variety of other pathologies can also be shown. It is occasionally difficult when looking at the thorax on the bone scan to determine whether a lesion is within bone or within the thoracic cavity. By using emission computed tomography to show the site of uptake and correlating this with transmission computed tomography an accurate display of both the anatomy and the pathology can be obtained.

The combined use of transmission and emission computed tomography demonstrate a correlative technique whereby a high degree of sensitivity and specificity may be obtained. Three cases are reported illustrating these points.

### CASE REPORT—(CASE A)

A 17 year old girl presented with a limp due to pain in her left knee. This was found to be due to an osteosarcoma of the upper tibia, which was surgically removed. Fourteen months later she developed pain in the right sacroiliac region, X-rays and CT of which showed a sclerotic region with some new bone formation. It was thought to represent either a metastatic deposit or a second primary osteosarcoma. A plain chest radiograph appeared normal but CT of the chest showed small pulmonary metastases and a bone scan planar image (Figure 1) showed areas of increased activity in the right sacroiliac area and over the chest. The latter area was related to a rib and it was therefore uncertain whether it represented a rib or intrapulmonary lesion. Emission computed tomography was performed (Figure 2) and this showed the increased uptake to be intrapumonary. A new CT scan (Figure 3) showed a centrally placed pulmonary metastatic deposit which had been previously missed due to the fact that it was interposed between blood vessels. The emission CT therefore revealed a lesion which had been previously missed on CT.

### CASE REPORT—(CASE B)

A 14 year old girl presented with pain and swelling in her left knee which investigation showed to be an osteosarcoma of the lower femur. This was excised, she was given a prosthetic insert and treated with methotrexate, adriamycin and cisplatinum. During follow up 1 year later a solitary pulmonary metastasis was seen on the chest X-ray (Figures 4a and b), following which radionuclide bone scanning and CT were performed. CT (Figures 5 and 6) showed there to be 3 lung metastases, the bone scan (Figure 7) showed an area of increased uptake in the left side of the chest related to one of the anterior ribs. It was uncertain whether this area corresponded to the known lung metastasis or represented a lesion in the rib. Emission computed tomography was therefore performed (Figure 8). This confirmed the area of increased uptake to correspond to the known lung metastasis and excluded the possibility of a rib lesion.

**Figure 1**

Planar image (anterior view) of isotope bone scan of Case A showing areas of increased activity over the right side of the pelvis and the anterior end of the right fourth rib.

**Figure 2**

ECAT image of isotope bone scan of Case A showing the area of increased activity in the thorax to lie posteriorly in the right lung rather than in a rib. (a: vertebral body, b: sternum, c: metastatic deposit).

**Figure 3**

Transmission CT section of Case A at the level of the lesion seen on the ECAT image showing a large metastatic deposit interposed between two of the lower lobe vessels.

**Figure 4**

(*above*) P.A. and (*below*) left lateral chest radiographs of Case B, showing a well defined calcific opacity in the left lower lobe, and a similar smaller lesion at the right base.

### CASE REPORT—(CASE C)

A 52 year old woman presented with a left breast carcinoma present for some months. This was removed surgically, a bone scan at the time being normal. Four years later she developed a persistent cough and chest X-ray showed bilateral hilar lymphadenopathy. The appearances were thought more typical and sarcoidosis than of metastatic deposits and there was some symptomatic improvement and shrinkage of the hilar nodes on steroid therapy. Three years later she developed shoulder pain and a radionuclide bone scane (Figure 9) was done to exclude the possibility of bony metastatic deposits. The images showed areas of increased uptake over the chest, thought possibly to lie with ribs. The appearances were however somewhat equivocal and emission CT was therefore performed (Figures 10a and b). This showed the areas of increased uptake to lie at both hila and not in the ribs. Transmission CT was then performed (Figure 11) and this showed the presence of calcification in the hilar nodes.

### DISCUSSION

Three cases are presented in which increased uptake of 99 m Tc methylene diphosphonate (MDP) was shown overlying ribs but further examination using emission computed tomography of the bone scan together with transmission computed tomography demonstrated the cause to be intrapulmonary. In two cases there was uptake in intrapulmonary metastatic deposits from osteosarcoma. In the third case there was uptake of MDP in calcified hilar nodes due to sarcoidosis.

The uptake of radiopharmaceuticals used for bone scanning by a variety of lung lesions is well recognised. Those

**Figure 5**

CT section of the mid-thoracic region of Case B showing a large, calcified metastatic deposit corresponding with the lesion on the chest radiograph and an additional smaller peripheral metastasis.

**Figure 6**

Transmission CT section of Case B just above the diaphragm showing the right basal metastasis seen on the chest radiograph.

**Figure 7**

Planar bone scan of Case B (anterior view) showing an area of increased activity in the region of the left fourth rib. There is absence of uptake at the site of resection in the left femur but increase at the proximal end.

reported include primary and secondary neoplasms (Poulose et al. 1975, Ghaed et al. 1981), radiation pneumonitis and one case of sarcoidosis (Poulose et al. 1975) and pulmonary emboli with coexistent hypercalcaemic (Wraight 1983). Previous reports also indicate that bone forming metastases may be detected by radionuclide scanning before becoming visible on the plain chest radiograph (McKillop et al. 1974, Robinson 1979). In this paper a case of MDP uptake in hilar nodes calcified due to sarcoidosis is reported – a combination that has only once previously been reported (Poulose et al. 1975). In some situations there is a possibility of both lung and bone lesions being present. The three cases presented all illustrate the difficulty which can be encountered in discriminating between such lesions using the standard planar bone scan images.

Emission computed tomography is a method by which a three dimensional image can be reconstructed from the distribution of a radiopharmaceutical administered to a patient. Its use with conventional radionuclide imaging of the skeleton using MDP has been limited but it is a useful method of localising areas of increased activity in the sites which are equivocal on planar images.

In all three cases presented the areas of increased activity in the thorax were localised in the lungs and rib lesions were excluded.

**Figure 8**

ECAT image of Case B at the level of the area of increased uptake seen on the planar image. It shows this to lie within the lung posteriorly rather than in a rib. (a: spine, b: sternum, c: metastatic deposit, d: ribs).

**Figure 9**

Posterior thoracic planar image from isotope bone scan of Case C, showing bilateral areas of increased activity over the medial ends of the posterior ribs.

**Figure 10(a) and (b)**

ECAT images of the hilar region of Case C showing the areas of increased uptake to lie at the hila and not in ribs. (a: spine, b: sternum, c: hilar lymph nodes).

**Figure 11**

Transmission CT image of the hilar regions of Case C, showing calcification in hilar lymph nodes bilaterally. There has been a left mastectomy.

Transmission computed tomography provides a fine display of anatomical detail and comparison of the emission CT images with those of transmission CT enables areas of increased activity to be identified with anatomical structures or pathological lesions (McMillan et al. 1983). This assists in determining the exact nature of the lesion, which is of prime importance for clinical management. The combination of the two techniques is therefore recommended in the evaluation of equivocal thoracic bone scan images.

### REFERENCES

1. GHAED, N., THRALL, J. H., PINSKY, S. M., JOHNSON, M. C. (1981) Detection of Extraosseous Metastases from Osteo sarcoma with 99mTc – Polyphosphate Bone Scanning. *Cancer* **48**, 113–1138.
2. McKILLOP, J. H., ETCUBANAS, E., GORIS, M. L. (1974) The Indications for and Limitations of Bone Scintigraphy in Osteogenic Sarcoma. A review of 55 patients. *Radiology* **112**, 373–375.
3. McMILLAN, P., JACKSON, P., DAVIES, E. R., GODDARD, P. (1983) Emission and Transmission Pulmonary Computed Tomography. *British Journal of Radiology* **56**, 991–992.
4. POULOSE, K. P., REBA, R. C., ECKELMAN, W. C., GOODYEAR, M. (1975) Extra-osseous localization of 99Tcm – Sn Pyrophosphate. *British Journal of Radiology* **48**, 724–726.
5. ROBINSON, P. J. (1979) Clinical Uses of Isotope Imaging. In *Medical Imaging,* Louis Kreel (ed.) England, HM + M Publishers, p. 228.
6. WRAIGHT, E. P. (1983) Focal lung uptake of technetium 99m methylene diphosphonate associated with pumonary emboli and hypercalcaemia. *British Journal of Radiology*, **56**, 345–349.

### ACKNOWLEDGEMENTS

The authors are grateful for the help from Miss J. Hugh, Dr. B. Hale and the Dept. of Medical Illustration.

## Figures and Tables

**Figure 1 f1:**
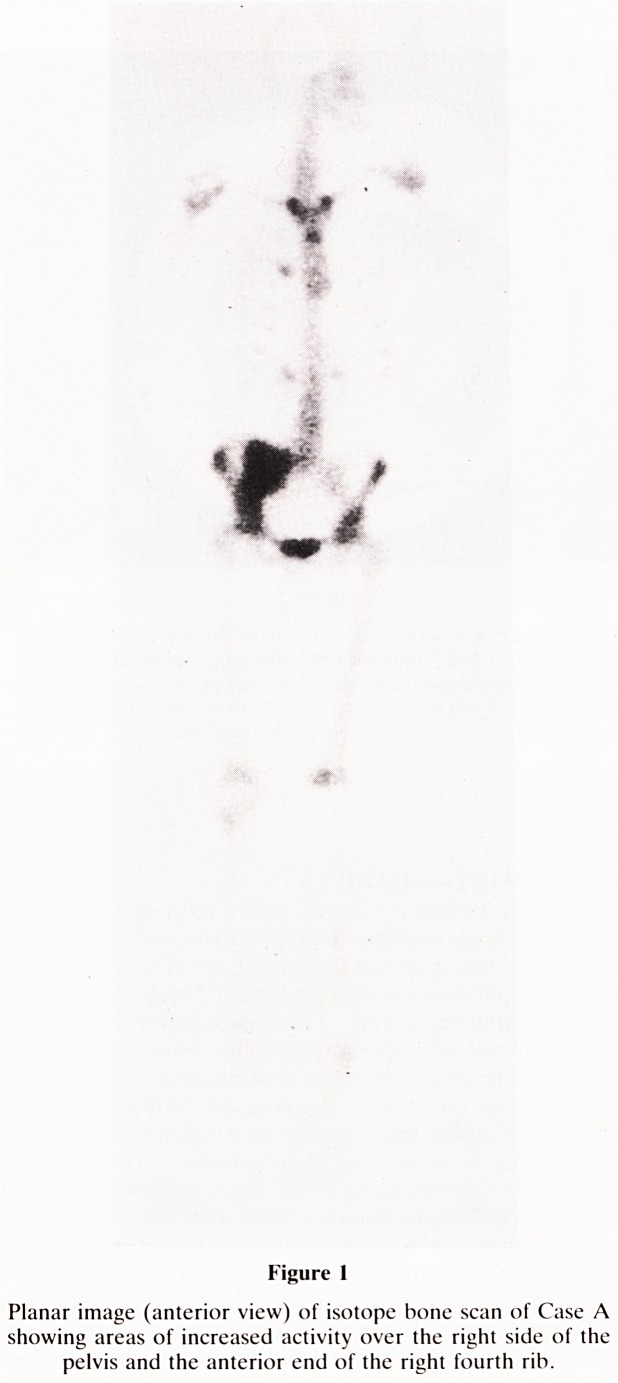


**Figure 2 f2:**
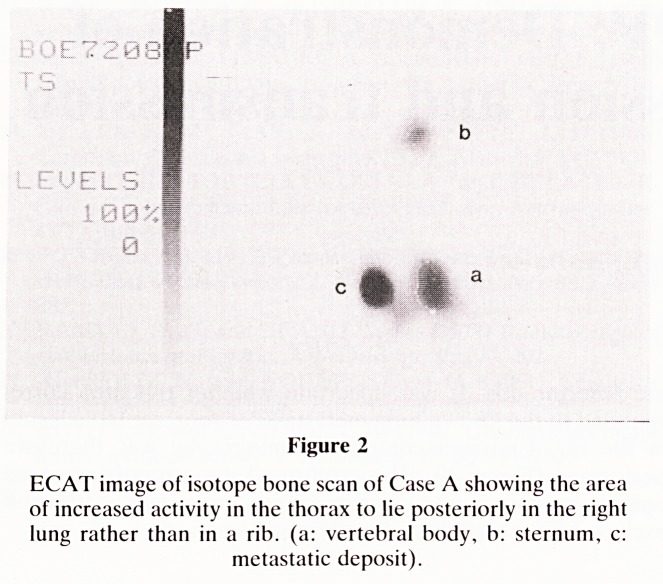


**Figure 3 f3:**
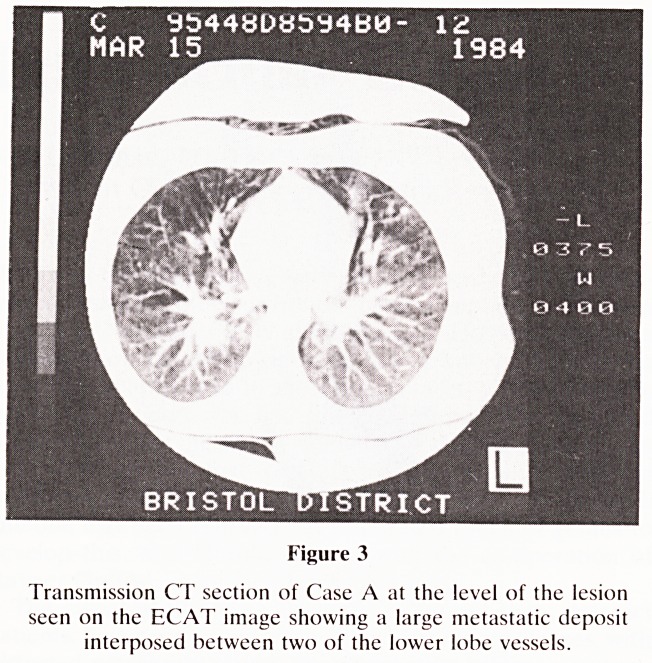


**Figure 4 f4:**
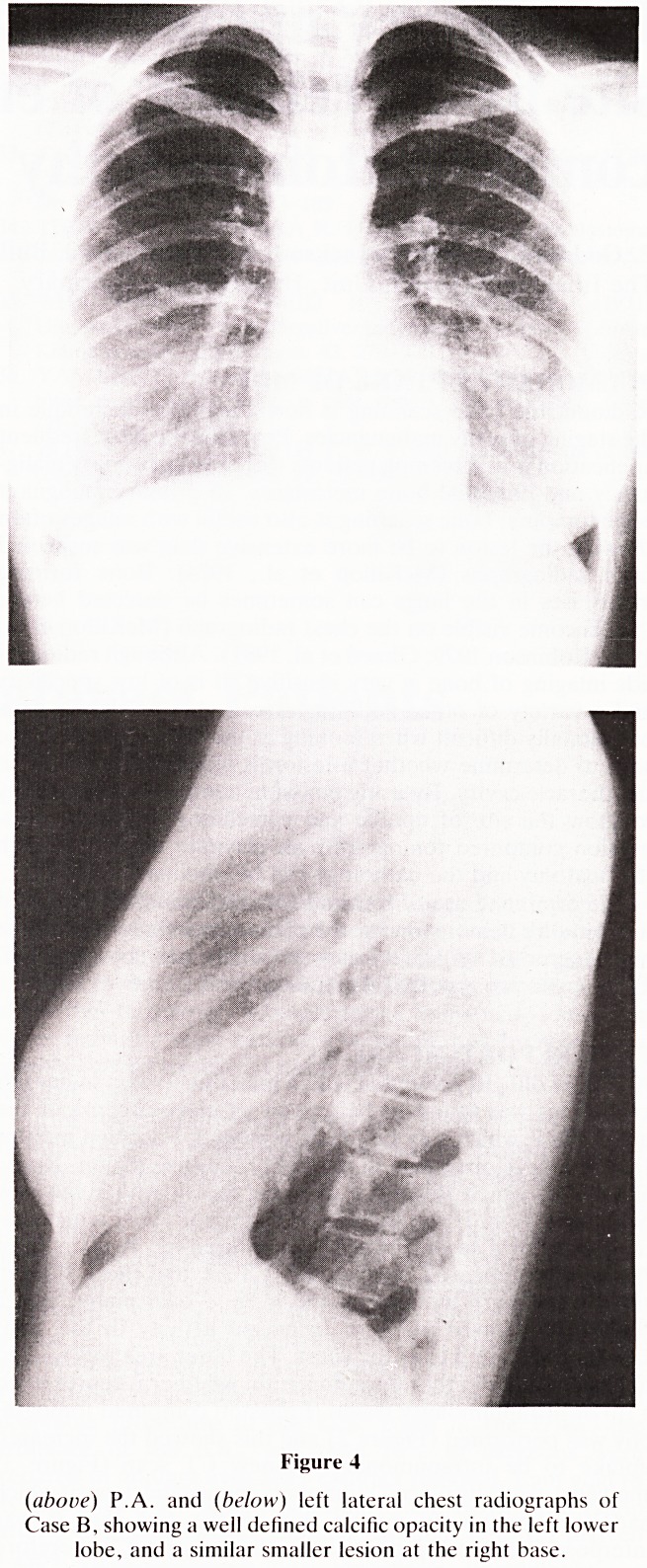


**Figure 5 f5:**
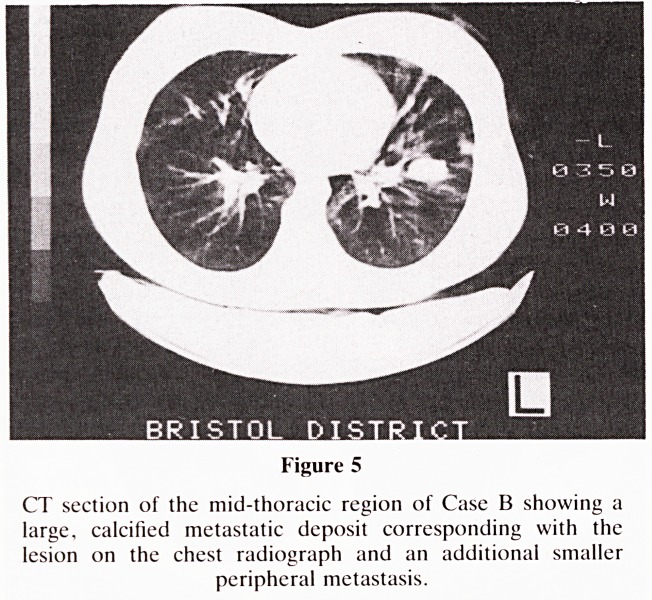


**Figure 6 f6:**
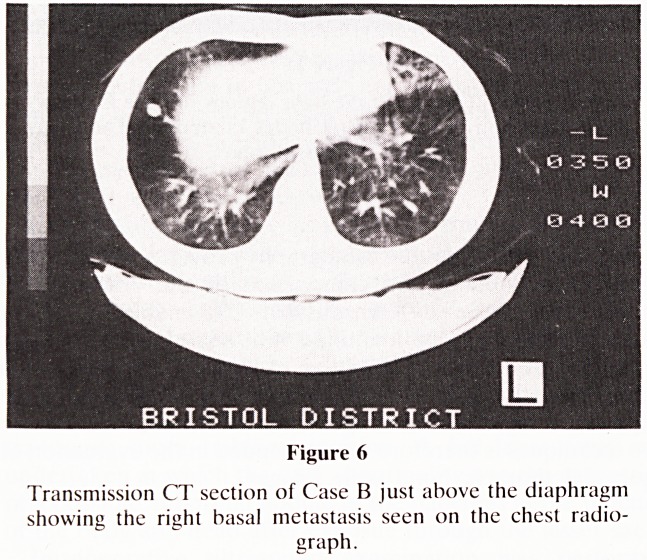


**Figure 7 f7:**
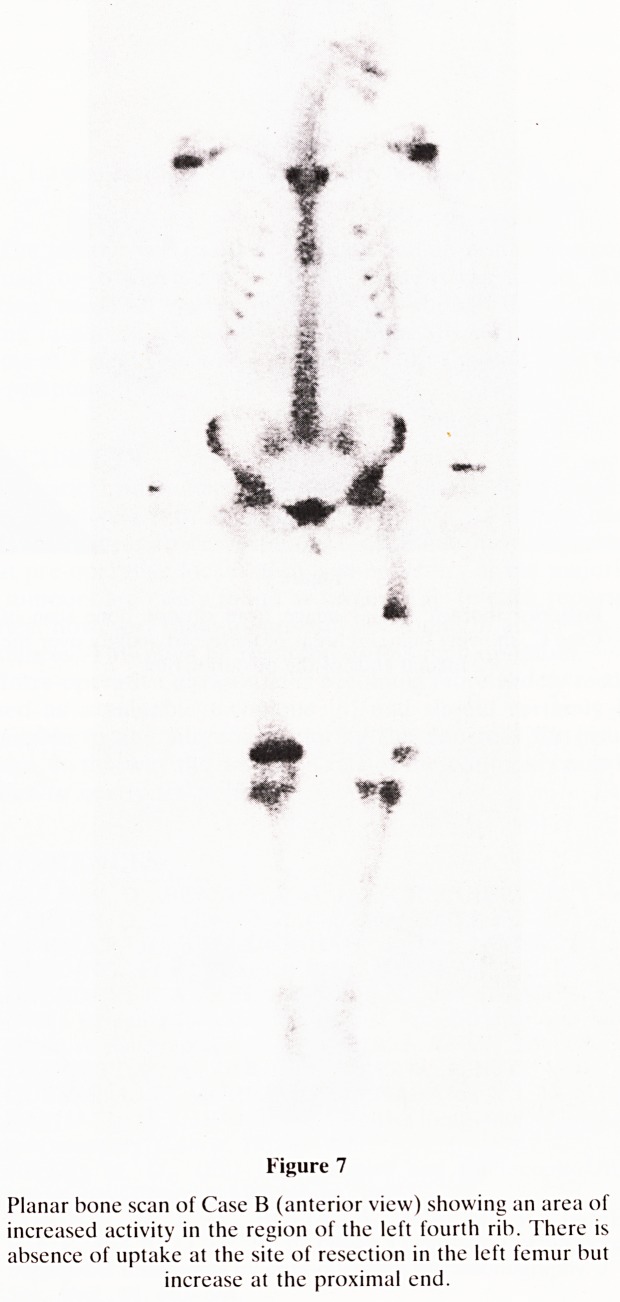


**Figure 8 f8:**
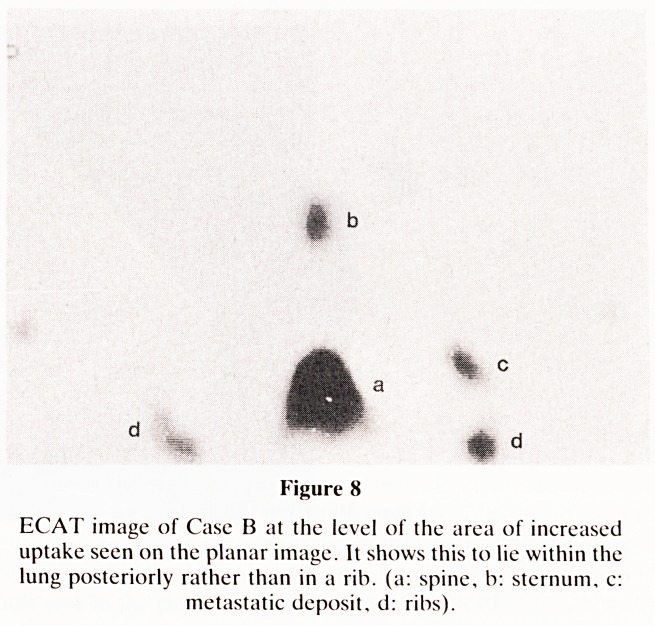


**Figure 9 f9:**
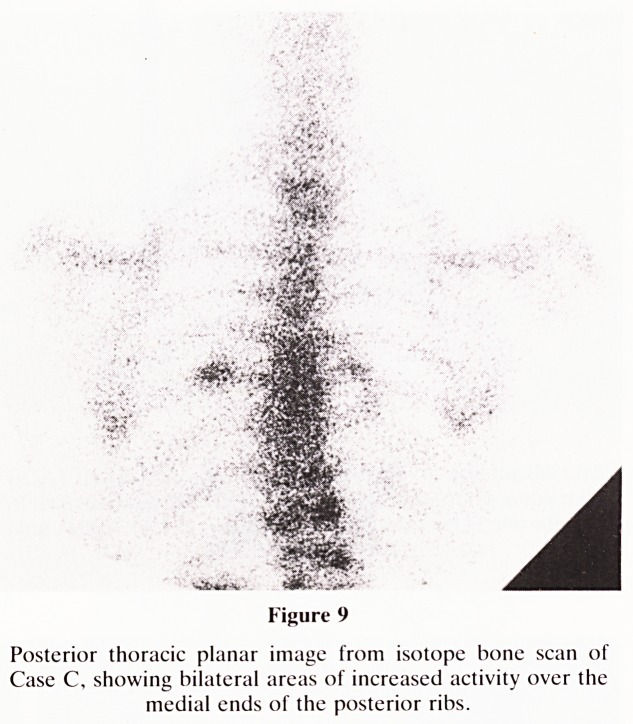


**Figure 10(a) and (b) f10:**
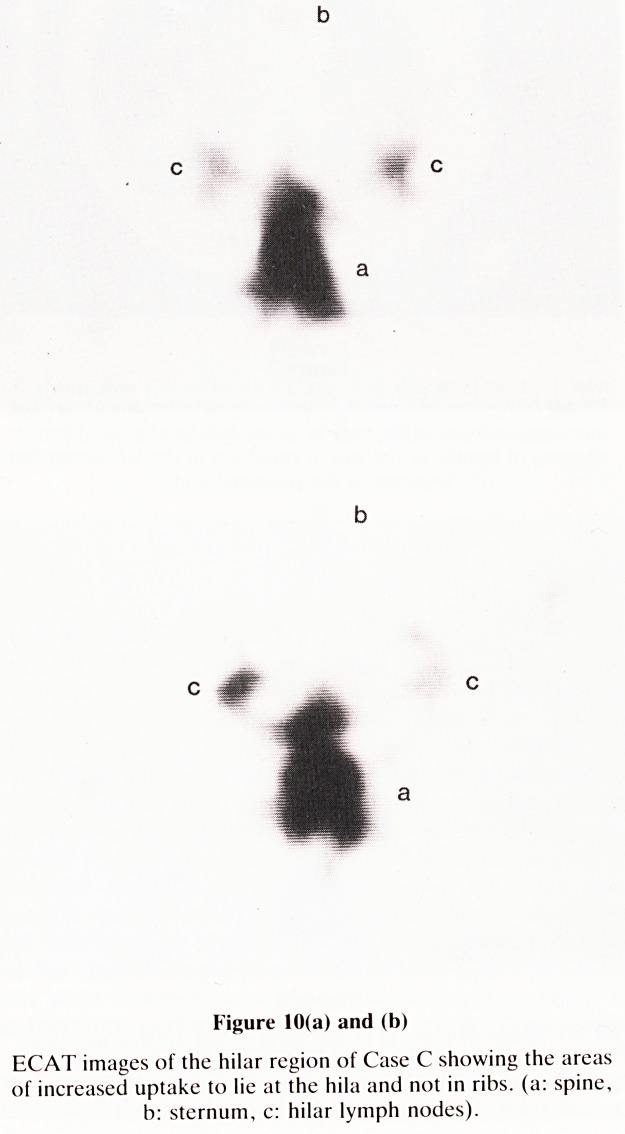


**Figure 11 f11:**